# MFAFENet: A Multi-Sensor Collaborative and Multi-Scale Feature Information Adaptive Fusion Network for Spindle Rotational Error Classification in CNC Machine Tools

**DOI:** 10.3390/e28040475

**Published:** 2026-04-20

**Authors:** Fei Wang, Lin Song, Pengfei Wang, Ping Deng, Tianwei Lan

**Affiliations:** 1 School of Low-Altitude Technology and Transportation, Chengdu Technological University, Yibin 644012, China; 2 School of Electrical Engineering, Southwest Jiaotong University, Chengdu 610031, China; 3 School of Mechanical Engineering, Chengdu Technological University, Chengdu 611730, China

**Keywords:** spindle, rotational error, multi-sensor, multi-scale feature information, attention mechanism

## Abstract

Accurate classification of spindle rotational errors is critical for ensuring machining precision and operational reliability of CNC machine tools. However, existing methods face challenges in extracting discriminative feature information from vibration signals due to small inter-class differences and complex electromechanical interference. This paper proposes a novel deep learning model, MFAFENet, based on multi-sensor collaboration and multi-scale feature information adaptive fusion. Vibration signals from three mounting positions are transformed into time-frequency information representations via Short-time Fourier Transform. The proposed network adaptively fuses multi-scale feature information from parallel branches with different kernel sizes through a branch attention mechanism. An efficient channel attention module is then incorporated to recalibrate channel-wise feature responses. The cross-entropy loss function is employed to optimize the network parameters during training. Experiments on a spindle reliability test bench demonstrate that MFAFENet achieves 93.37% average test accuracy, outperforming other comparative methods. Ablation and comparative studies confirm the effectiveness of each module and the clear advantage of adaptive fusion over fixed-weight multi-scale methods. Multi-sensor fusion further improves accuracy by 7.23% over the best single-sensor setup. The proposed method establishes an effective end-to-end mapping between vibration signals and rotational errors, providing a promising solution for high-precision spindle condition monitoring.

## 1. Introduction

CNC machine tools are essential core equipment in modern manufacturing [1]. Through digital programming, precise control of the machining process is achieved, enabling high-precision, high-efficiency, and mass production of complex components. Whether it is high-strength alloy components in the aerospace industry [2], precision engine components in the automotive industry [3], or artificial implants in the medical industry [4], CNC machine tools can ensure micrometer level machining consistency, significantly reducing human error and production costs. The technological level of CNC machine tools directly determines the competitiveness of a country’s high-end manufacturing industry and is an important cornerstone for promoting industrial upgrading and technological innovation. The spindle of a CNC machine tool is the core power component, which directly drives the tool or workpiece for high-speed and precision rotary cutting. It consists of high-precision bearings, motors, and cooling systems, supporting various processes such as high-speed machining and heavy-duty cutting. The precision errors of various parts of the machine tool are ultimately directly displayed on the spindle, the performance of the spindle directly determines the machining accuracy, surface quality, and efficiency of the machine tool, and is the technical bottleneck and value core of high-end manufacturing equipment.

The important indicator for quantitatively measuring the performance of the spindle is the rotational error [5]. The rotational error of a CNC machine tool spindle refers to the maximum deviation of its actual rotational axis from the ideal rotational axis in spatial position during the rotation process. As the rotational error increases, the circular trajectory at the tip of the spindle tool transitions from a smooth circle to an irregularly deformed shape with concavities and convexities [6]. Accurately measuring the spindle rotational error not only enables monitoring of the spindle’s operating status, facilitating timely detection and diagnosis of faults, but also allows for error compensation to enhance rotational accuracy. Furthermore, it helps prevent excessive centrifugal forces caused by increased rotational error, thereby mitigating potential safety risks to operators [7,8].

Through a review of existing literature, it has been found that spindle rotation error measurement methods can be categorized into physical measurement methods, physical modeling methods, and data modeling methods. Physical measurement methods, such as the reversal method, multi-point method, and their variants [9,10,11,12,13] typically rely on contact or non-contact displacement sensors, standard artifacts (e.g., standard balls or bars), and precise indexing mechanisms. While these methods can achieve high accuracy under controlled conditions, they are generally unsuitable for online measurement during actual machining operations due to their intrusive nature, dependence on tool-occupying artifacts, susceptibility to installation errors, and inability to account for system vibrations under varying loads. Consequently, they struggle to meet the real-time and in-process requirements of CNC machine tools under high-speed cutting conditions. Physical modeling methods, on the other hand, focus on analyzing the dynamic characteristics of spindle systems. Researchers have developed various dynamic models incorporating factors such as shaft roundness, cylindricity, unbalanced forces, and cutting loads to simulate spindle rotational errors [14,15,16]. However, these methods are fundamentally constrained by the oversimplification of complex spindle dynamics, which limits their accuracy in representing real-world machining processes. Moreover, constructing high-fidelity dynamic models is time-consuming, labor-intensive, and requires extensive physical expertise, significantly hindering their practical application in industrial settings [10].

With the rapid advancement of sensor technology, data-driven modeling methods using vibration signals have gained significant traction. Vibration sensors are widely deployed in mechanical system monitoring [17,18], and the vibration signals of a spindle are rich in information related to its operational state [19,20]. Early data-driven attempts utilized manual feature extraction, such as information entropy, which struggles to capture complex degradation trends [21,22]. Subsequently, researchers successfully employed neural networks, including LSTM and ResNet, to map vibration signals to rotational errors [23,24,25]. While these methods have achieved initial success, a critical distinction must be made: unlike fault diagnosis where inter-class differences are typically pronounced, rotational error classification is a non-fault task characterized by extremely small inter-class distances and high feature similarity. Furthermore, the spindle is a complex electromechanical system where vibration signals are susceptible to significant noise and interference, making the extraction of discriminative features a formidable challenge.

To extract important feature information from vibration signals, Song et al. developed a rotational error classification method based on multi-scale feature information extraction using convolutional neural networks [26]. In the context of vibration signal-based condition monitoring tasks, Peng et al. proposed a fault diagnosis method employing a multi-branch and multi-scale convolutional neural network (CNN) [27]. Song et al. introduced multi-scale grouped convolution for bearing fault diagnosis [28]. Du et al. utilized continuous wavelet transform combined with a multi-scale CNN to achieve deep feature extraction for bearing remaining useful life prediction [29]. These multi-scale feature information extraction methods follow a similar principle: they employ multiple branches, each using convolutional kernels of different sizes to extract features, and finally concatenate or sum the results from these branches [21]. However, these methods cannot dynamically adjust the weights of different convolutional branches, and the feature information extraction potential of multi-scale convolutional networks has not been fully exploited. However, a review of the current state-of-the-art reveals two critical and unresolved limitations:

(1) Inefficient Multi-Scale Fusion: Existing multi-scale structures typically fuse the outputs of different branches by simple concatenation or summation with fixed weights. They fail to model the interrelationships between branches, preventing the network from dynamically adjusting the importance of different scales based on the input signal. This leads to redundant information propagation and suboptimal feature extraction.
(2) Lack of Channel-Wise Modeling: After multi-scale fusion, there is no efficient mechanism to model the interdependencies among feature channels. This oversight means the network cannot automatically prioritize more important channel information while suppressing less relevant ones, limiting its discriminative power for tasks with small inter-class differences.

In summary, while data-driven methods represent a promising direction for spindle rotational error classification, existing multi-scale CNNs are hindered by static fusion strategies and a lack of channel-wise feature recalibration. To address these issues, this paper adopts the multi-scale feature adaptive fusion convolutional (MFAFConv) module, which is inspired by the Selective Kernel Network [30] architecture, and adapts it to the spindle rotational error classification task. Unlike the original SKNet, which uses dilated convolution, the MFAFConv module employs depthwise separable convolution in each branch, significantly reducing model parameters while maintaining expressive power. The MFAFConv module dynamically aggregates multi-scale feature information by modeling interrelationships among different convolutional branches and assigning adaptive weights to each branch. Furthermore, an efficient channel attention (ECA) mechanism is incorporated after multi-scale feature fusion to recalibrate channel-wise feature responses. Building upon these components, this paper proposes a deep learning model for spindle rotational error prediction, namely the multi-scale feature information adaptive fusion and enhancement network (MFAFENet). MFAFENet takes vibration sensor signals from three different positions of the spindle system as input, converts them into time-frequency feature information via short-time Fourier transform (STFT), and then employs multi-scale feature adaptive fusion convolutional (MFAFConv) layers to extract key feature information. An enhancement module is further utilized to highlight feature information from important channels. After processing through multiple MFAFConv layers and enhancement modules, the high-dimensional feature information are fed into a global average pooling (GAP) layer and a fully connected layer to obtain the rotational error prediction results. MFAFENet can establish an end-to-end mapping relationship between spindle vibration signals and rotational errors, and can automatically adapt to multi-scale feature information based on different input signals. The core innovations of this paper can be summarized as follows:

(1) This paper introduces the MFAFConv module to the spindle rotational error classification task. Unlike the original SKNet, MFAFConv employs depthwise separable convolution in each branch, which significantly reduces model parameters while maintaining expressive power. It extracts multi-scale feature information from vibration signals, models interrelationships among different scale branches, and adaptively adjusts branch weights to enhance feature representation.
(2) An efficient channel attention (ECA) mechanism is incorporated after the multi-scale feature fusion stage to model channel-wise dependencies, which helps prioritize informative channels and suppress less relevant ones. The combination of MFAFConv and ECA provides both multi-scale adaptive fusion and channel-wise recalibration.
(3) The MFAFConv layer and ECA module are integrated into a ResNet architecture to construct MFAFENet. Experimental results on a spindle reliability testbed demonstrate that this combination improves the accuracy of spindle rotational error classification compared to several baseline methods.

The remaining content is structured as follows: Section 2 introduces the ResNet architecture, which serves as the foundation of MFAFENet; Section 3 provides a detailed description of the presented MFAFConv layer and the ECA enhancement module, followed by the construction of MFAFENet based on these components; Section 4 presents the spindle reliability experimental platform and validates the effectiveness and superiority of the proposed method using data collected from this platform; Section 5 summarizes the full text and draws conclusions.

## 2. ResNet

**Figure 1:**
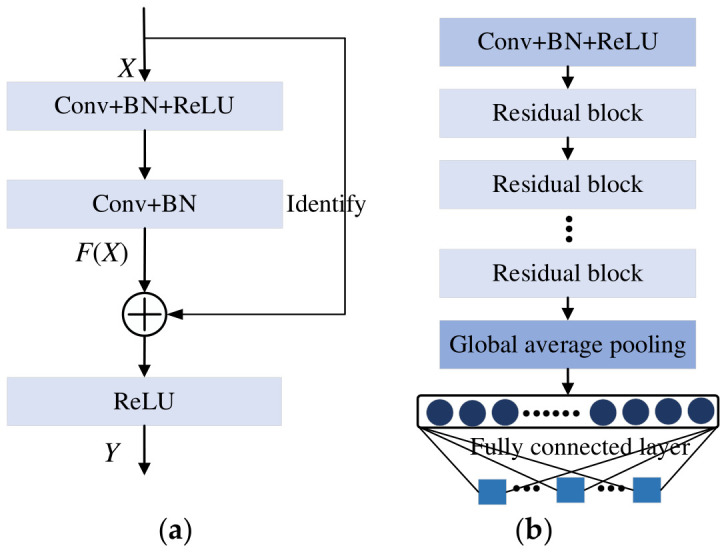
Schematic diagram of residual network structure. (**a**) Basic residual block consisting of convolutional layers, batch normalization, and ReLU activation with an identity shortcut connection; (**b**) Overall architecture of ResNet constructed by stacking multiple residual blocks.

As the backbone of the proposed MFAFENet, the ResNet architecture is briefly introduced here. To overcome the optimization challenges in deep networks, He et al. [31] proposed ResNet with a residual learning mechanism. Its core idea is to introduce a residual learning mechanism, whereby the network no longer directly learns the complex underlying mapping H(x), but instead learns the residual F(x)=H(x)−x between that mapping and the input x. By adding the input x to the learned residual F(x) via an identity mapping, the final output F(x)+x is obtained. This design effectively alleviates the vanishing gradient problem, enabling the training of very deep networks and significantly enhancing feature extraction and representation capabilities. The residual block, as shown in Figure 1, typically consists of convolutional layers, batch normalization (BN) layers, and ReLU activation layers. In the figure, ⊕ represents the summation operation. In this study, we will enhance this residual block with our proposed MFAFConv and ECA modules to better suit the spindle rotational error classification task. 

## 3. The Proposed MFAFENet Prediction Method

Existing multi-scale CNNs for spindle rotational error classification typically extract feature information using parallel convolutional branches with different kernel sizes and then fuse them through concatenation or summation. Although this strategy can capture multi-scale time–frequency information from vibration signals, it still suffers from two key limitations. First, the relationships among different scale branches are not explicitly modeled, and the fusion process usually assigns equal or fixed weights to all branches, making it difficult for the network to adaptively emphasize more informative scales under varying operating conditions. This often leads to redundant feature information propagation and weakens the discriminative capability required for rotational error classification, where inter-class differences are inherently small. Second, after multi-scale feature information fusion, effective modeling of channel-wise dependencies is usually lacking, which prevents the network from automatically highlighting channels that are more relevant to rotational errors while suppressing less informative or noise-dominated channels. To address these issues, this paper proposes MFAFENet, which integrates a multi-scale feature adaptive fusion convolutional layer (MFAFConv) and a lightweight ECA enhancement module into a ResNet framework, enabling adaptive multi-scale feature information weighting and channel-wise feature information recalibration, and thereby improving the robustness and accuracy of spindle rotational error classification.

### 3.1. MFAFConv Module

**Figure 2:**
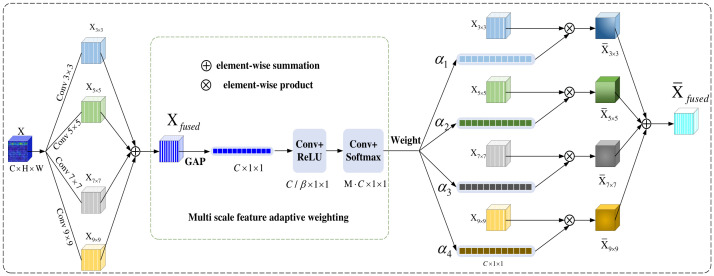
Architecture of the proposed multi-scale feature adaptive fusion convolutional (MFAFConv) module. The module uses four parallel branches with kernel sizes of 3 × 3, 5 × 5, 7 × 7, and 9 × 9, followed by a branch attention mechanism for adaptive weight assignment.

In order to dynamically aggregate feature information with different receptive fields within a single convolutional module, enabling the network to adaptively adjust the receptive field size based on the input content and thus enhance the model’s ability to capture multi-scale information, this paper proposes a multi-scale feature adaptive fusion and enhancement network (MFAFENet) for the prediction of CNC machine tool spindle rotational error. The receptive fields of neurons in the human visual cortex can be dynamically adjusted based on the characteristics of input stimuli, a physiological mechanism that provides important inspiration for multi-scale feature information extraction in computer vision. Inspired by this, the MFAFConv module is designed with the core idea of deploying parallel branches with different convolutional kernel sizes to simultaneously capture multi-scale time-frequency feature information, thereby enhancing the network’s adaptability to environmental changes. During the information fusion stage, the module introduces an attention mechanism to adaptively assign appropriate weights to different convolutional branches according to the input content, thereby highlighting the scale feature information more critical to the current task while suppressing redundant or interfering information. This structure improves the model’s robustness in recognizing different vibration signals and the discriminability of feature representations. Specifically, as shown in Figure 2, the MFAFConv module (illustrated with a four-branch structure) compresses global information via global average pooling, models inter-branch relationships through two convolutional layers, and generates adaptive weights via Softmax. This provides a principled way to aggregate multi-scale features rather than using fixed concatenation or summation.

Suppose the time-frequency coefficient matrix obtained after processing a one-dimensional vibration signal by STFT be denoted as $X\text{∈}R^{C\times H\times W}$, where C represents the number of channels, H denotes the height of the two-dimensional time-frequency coefficient matrix, and W denotes the width. Passing X through four convolutional layers with different kernel sizes (specifically 3 × 3, 5 × 5, 7 × 7, and 9 × 9) yields the corresponding outputs $X_{3\times 3}$, $X_{5\times 5}$, $X_{7\times 7}$, and $X_{9\times 9}$. Notably, depthwise separable convolution is adopted in each branch during the convolution operation, where the input signal is divided into C groups (the number of groups equals the number of input channels). This design significantly reduces the number of parameters in the model. Additionally, a Conv → BN → ReLU structure is employed.

As analyzed earlier, the objective of this paper is to enable the model to adaptively fuse multi-scale feature information according to different vibration signals, thereby achieving the goal of automatically adjusting the receptive field. The core idea is to integrate feature information from different branches and propagate it to the next layer of neurons.

**Multi-branch fusion information extraction:** First, the feature information from different branches is integrated using element-wise summation:

(1) $$X_{fused}=\sum_{m=1}^{M}X_{m}$$

where $X_{fused}$ represents the result of element-wise summation of the feature information from the four branches. $X_{m}$ represents the output of the m-th branch convolutional layer with a kernel size of (2m+1). M denotes the number of branches. Then, to establish dependencies among different branches, the fused global information is compressed into channel descriptors. This process is achieved through global average pooling, which can be expressed as:

(2) $$X_{GAP}=\frac{1}{H\times W}\sum_{i=1}^{H}\sum_{j=1}^{W}X_{fused}^{c}(i,j)$$

where $X_{GAP}\in C\times 1\times 1$ represents the channel descriptor, which is a statistical measure capable of characterizing the global information of features from branches at different scales.

**Multi-branch relationship modeling:** furthermore, to recalibrate the importance of different convolutional kernel branches, thereby enabling the model to dynamically aggregate feature information from receptive fields of varying sizes, this step must be capable of learning nonlinear interactions between the branches. This is achieved through a 2D convolutional (Conv) followed by a ReLU nonlinear transformation. The input feature information $X_{GAP}$ in this step has the dimension C×1×1, with C representing the number of channels. A convolution operation with a kernel size of 1×1 is applied to the feature to integrate information across different channels, thereby enabling multi-branch relationship modeling. To reduce the computational cost of the model, a reduction factor β is used to compress the number of output channels of this Conv, making it smaller than the number of input channels. Specifically, the convolution employed for multi-branch relationship modeling takes C input channels and reduces the output channels to C/β. Subsequently, a ReLU activation function is applied to incorporate nonlinearity to the output features. This process can be expressed as:

(3) $${X_{Conv1}=ReLU(W}_{1}*X_{GAP})$$

where $W_{1}\in R^{(C/\beta )\times C}$ represents the weight of the first convolutional layer.

**Weight generation:** The weights of each branch are generated through another convolutional layer and softmax function, thereby adaptively selecting the spatial scales of different information. The input channels of the second convolutional layer are C, and the output channels are M∗C. Then, through a Reshape operation, the dimension of the output matrix is transformed into M×C. This process can be expressed as:

(4) $${z=Reshape(W}_{2}*X_{Conv1})$$

where $W_{2}\in R^{(M*C)\times C/\beta }$ represents the weight of the second convolutional layer. $z\in R^{M\times C}$ represents the output of the second convolutional layer. The Softmax operation is applied to z along the branch dimension to obtain the weight $a_{m}$ for each branch.

(5) $$a_{m,c}=\frac{e^{z_{m,c}}}{\sum_{m^{\prime }=1}^{M}e^{z_{m^{\prime },c}}}$$

Thus, for different input samples and different branches, each channel has a corresponding weight, and the sum of weights along the branch dimension is 1, $\sum_{m^{\prime }=1}^{M}a_{m^{\prime },c}=1$. Finally, the weights are divided into M groups according to the number of channels, with the weights for each group being $a_{m}\in R^{C\times 1\times 1}$. Taking four branches as an example, the final fused output ${\bar{X}}_{fused}$ after weighted fusion with various convolutional kernels can be expressed as:

(6) $${\bar{X}}_{fused}=\sum_{m=1}^{M}{a_{m}⊗X}_{m}$$

where ⊗ denotes element-wise product.

### 3.2. Efficient Channel Attention Module

The MFAFConv layer models dependencies among multi-scale feature information along the branch dimension, enabling adaptive allocation of branch weights and dynamic aggregation of feature information from varying receptive fields. However, after feature information fusion, explicit modeling of feature dependencies along the channel dimension is absent, which prevents the evaluation of importance differences among channels in the fused feature information. In convolutional operations, although each filter can adaptively extract input feature information and map them to output channels, existing methods treat all channel feature information as equally important by default. This overlooks the varying contributions of different channels to the final prediction, thereby limiting feature utilization efficiency. To address this limitation, this study introduces an Efficient Channel Attention (ECA) module after the MFAFConv layer. This module models inter-channel dependencies along the channel dimension, allowing the network to dynamically identify and enhance critical feature channels while suppressing redundant or less important channel information. This adaptive channel feature information recalibration mechanism enables the network to strengthen more discriminative channel feature information for the task of rotation error classification, thereby improving the discriminative power and representational efficiency of multi-scale feature learning.

**Figure 3:**
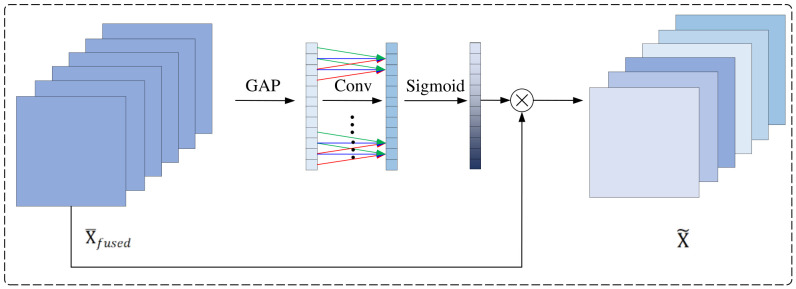
Architecture of the efficient channel attention (ECA) module. The module compresses spatial information via global average pooling, models channel-wise dependencies using a 1D convolution with adaptive kernel size, and generates channel attention weights via a Sigmoid function. In the figure, ⊗ represents assigning a weight to each channel, and all values of each channel are multiplied by the corresponding weight.

Illustrated in Figure 3, as the MFAFConv module compresses the fused information into channel descriptors, the ECA module performs a similar operation by compressing the statistical information of each channel through GAP. Then, a convolutional layer is used to establish interdependencies among the channels. Finally, the Sigmoid activation function is applied to generate weights for each channel. The output of the Sigmoid activation function ranges between 0 and 1, ensuring that the feature information from multiple channels is emphasized. This process can be expressed as:

(7) $${\tilde{X}=\bar{X}}_{fused}⊗(Sigmoid(Conv(\frac{1}{H\times W}\sum_{i=1}^{H}\sum_{j=1}^{W}{\bar{X}}_{fused}\left(i,j\right))))$$

where $\tilde{X}$ represents the feature information recalibrated along the channel dimension. It is worth noting that in the above process, the convolutional layer used to establish cross-channel interactions has both input and output channels set to 1, and its kernel size k is determined by the number of channels in the feature map. This is an extremely lightweight operation, introducing only k parameters to the network—an amount negligible compared to the parameter count of ResNet. The kernel size k follows the calculation method described in the literature [32], and can be expressed as:

(8) $$k={\left|\frac{{log}_{2}(C)+b}{r}\right|}_{odd}$$

where r and b are set to 2 and 1, respectively. ${\left|t\right|}_{odd}$ denotes the nearest odd integer to t. 

### 3.3. MFAFENet

**Figure 4:**
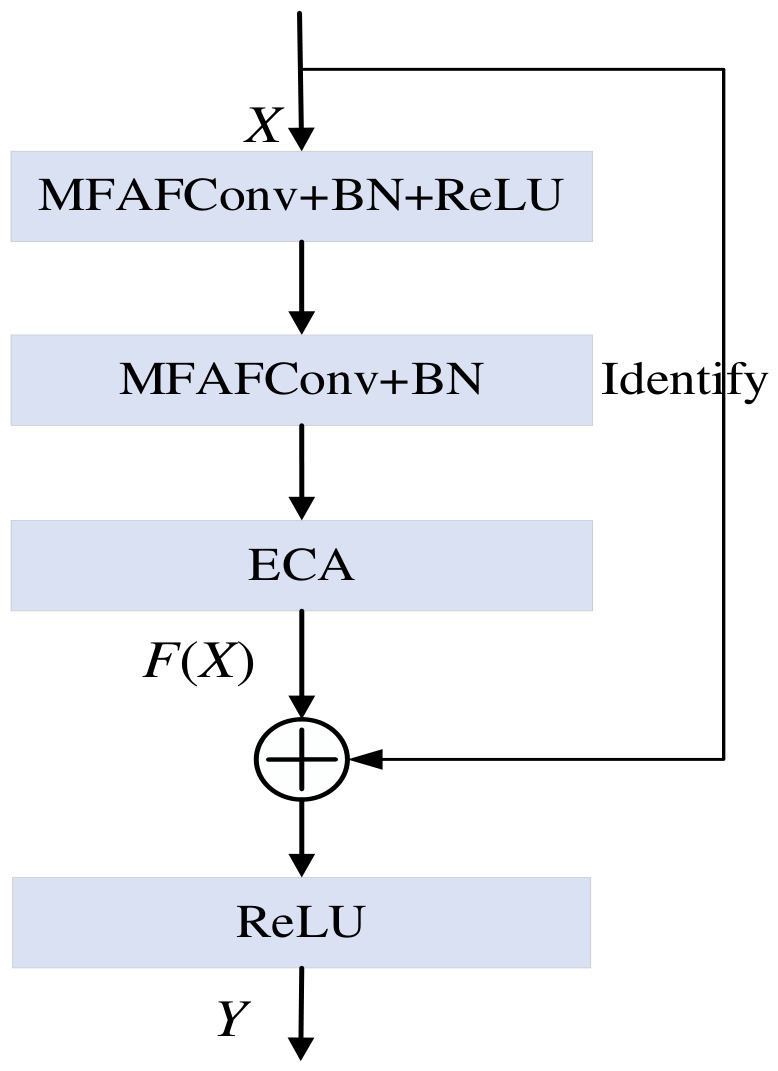
Structure of the improved residual block in MFAFENet. The standard convolutional layer in the original ResNet block is replaced with the MFAFConv module, and an ECA module is added after the second batch normalization layer to recalibrate channel-wise feature responses.

As shown in Figure 4, replacing the original Conv module in the standard ResNet of Figure 1 with the MFAFConv module and adding the ECA module after the second BN layer yields the improved residual block of MFAFENet. By stacking multiple such enhanced residual blocks, followed by a GAP layer and a fully connected layer, the MFAFENet architecture is constructed. The detailed model structure of MFAFENet is presented in Table 1. In the table, the convolutional stride in the Conv + BN + ReLU layers is 2 × 2, while within the improved residual block it is 1 × 1. The notation “Improved residual block × 2” indicates that two identical residual blocks are used. 3, 5, 7, 9 indicate that the convolutional kernels of the four branches in the Improved residual block are 3×3, 5×5, 7×7, and 9×9 respectively.

### 3.4. Cross-Entropy Optimized MFAFENet

**Figure 5:**
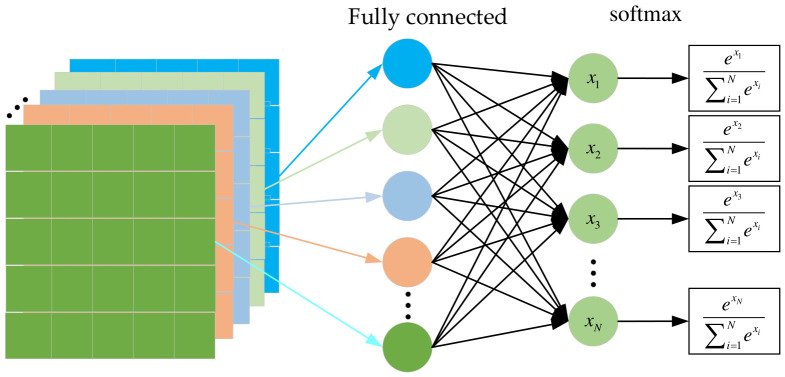
Schematic diagram of softmax calculation.

The loss function is employed to evaluate the error between the model’s predicted values and the true values, thereby guiding the model to optimize in the direction of error reduction. In this paper, the loss function employed for the multi-class classification task is the cross-entropy loss function. In multi-class classification tasks, sample labels are encoded using one-hot, where the label for each category is defined as a vector. In this vector, only one element has a value of 1, and the rest are 0. The length of the vector equals the number of categories, and the position of the value 1 indicates the specific category. Before calculating the cross-entropy loss, the output of the fully connected layer in Table 1 needs to be transformed by the softmax function, which converts the neuron outputs into values between 0 and 1, with the sum of all values equaling 1. The softmax function can be expressed as:

(9) $$p_{j}=\frac{e^{x_{j}}}{\sum_{i=1}^{N}e^{x_{i}}}$$

where N represents the number of categories; $x_{i}$ denotes the output of the fully connected layer; and $p_{j}$ represents the output of $x_{j}$ after softmax. Figure 5 shows a schematic diagram of the softmax calculation. Finally, the model loss is calculated using the cross-entropy loss function. Under one-hot encoding, the cross-entropy loss function can be expressed as:

(10) $$L_{CEL}=-\sum_{k=1}^{N}r_{k}log{(p}_{k})$$

where $r_{k}$ is the true value; $p_{k}$ represents the output after softmax, also known as the predicted value; and $L_{CEL}$ denotes the cross-entropy loss.

## 4. Experimental Verification

A large amount of data was collected from a CNC machine tool spindle reliability test bench to validate the prediction performance of the proposed method. This includes experiments on MFAFENet effectiveness, ablation study, selection of multi-scale branch number, multi-scale feature learning comparisons, and multi-sensor comparisons. All network implementations were coded in Python 3.11 using the PyTorch 2.22 framework, and experiments were conducted on a Windows system equipped with an i7 CPU and an RTX 2060 SUPER GPU.

### 4.1. Experimental Platform and Dataset

**Figure 6:**
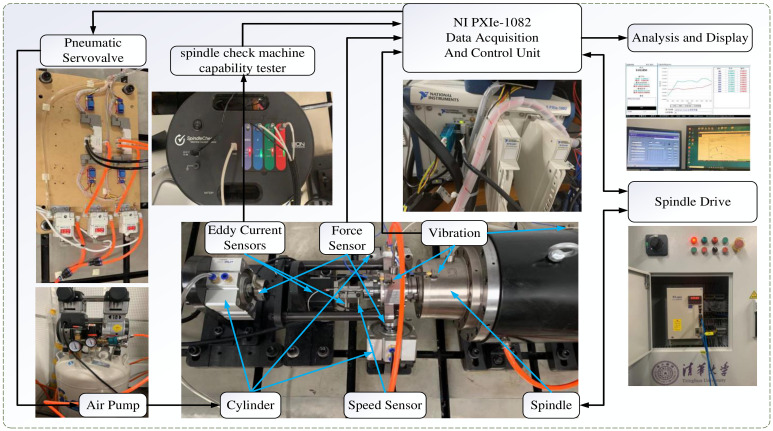
Experimental platform for spindle rotational error and vibration signal acquisition, including the pneumatic loading module (air pump, servo valves, cylinder), control module (PXIe-1082 controller), and data acquisition module (vibration sensors, force sensors, and Lion Precision rotational error measurement system).

As shown in Figure 6, this paper establishes an experimental platform for predicting the rotational error of machine tool spindles. The platform primarily consists of three parts: pneumatic loading, control, and data acquisition. In the pneumatic loading module, servo valves, an air pump, and a cylinder work in coordination: three sets of servo valves are used for precise air pressure regulation, the air pump provides a stable air supply, and the cylinder (with a diameter of 100 mm and a maximum theoretical loading force of 4000 N) converts air pressure into mechanical force, which acts on the loading mechanism. During the experiment, loads are applied according to the spindle load spectrum to simulate actual machining conditions [33]. The load spectrum is derived from a probability distribution model and discretized into intervals to generate a list of loading forces with corresponding frequencies or durations. The construction procedure is as follows: the load spectrum is divided into 8 intervals for both the mean load and the amplitude load. The mean load spectrum uses equally spaced intervals with proportional coefficients of 0.125, 0.250, 0.375, 0.500, 0.625, 0.750, 0.875, and 1.000. The amplitude load spectrum uses unequally spaced intervals with proportional coefficients of 0.125, 0.275, 0.425, 0.575, 0.725, 0.850, 0.950, and 1.000. Based on these interval divisions, the probability (cycle count) for each interval is determined. The detailed tangential force loading spectrum can be found in the literature [33]. During loading, the inner ring of the HRB 71909CTA bearing rotates with the spindle, while the outer ring remains stationary via a fixed sleeve. Specifically, the spindle used in this platform is a built-in motorized spindle with a BT40D tool interface (model: BT40D-4013-1800/12000), which integrates the motor and shaft as a single unit for direct drive operation without belts or gears. The spindle has a rated power of 13 kW and a maximum continuous speed of 12,000 r/min. The bearing system consists of angular contact ball bearings with permanent grease lubrication and a factory-set constant preload suitable for high-speed operation.

The control module centers around National Instruments’ PXIe-1082 data acquisition and control unit, integrating components such as spindle drive and analysis display. It is responsible for controlling the air pump loading, synchronizing data acquisition from multiple sensors, transmitting data, and providing spindle motion feedback. The PXIe-1082 communicates with the spindle driver via an RS485-USB interface to achieve precise control of spindle torque and rotational speed. The data acquisition module includes three types of sensors: three PCB 256A14 vibration sensors (positioned at the bearing end, spindle end, and base end, respectively, connected to the system via BNC interfaces), three DYMH-104 force sensors (connected to the analog input ports of the PXIe-1082 via four-wire differential signals to monitor loading forces in real-time), and a rotational error testing system developed by Lion Precision. This system integrates three eddy current sensors (for X, Y, and Z axial displacement acquisition), one speed sensor (for monitoring spindle rotational speed), and a dedicated conditioning converter. It communicates with the PXIe-1082 via a USB interface to perform online calculation of rotational errors. The platform provides a foundation of multi-physical, high-synchronization experimental data for subsequent method validation.

During the experiment, the rotational error measuring instrument collected the spindle’s rotational error and the corresponding vibration signals at 10 h intervals. A daily 10 h loaded wear test was conducted to induce gradual performance degradation in the rotating machinery. The experiment covered four rotational speed conditions: 1000 r/min, 2000 r/min, 3000 r/min, and 4000 r/min. Rotational errors and vibration signals under these different speeds were collected while the spindle operated under combined conditions of varying speed and load. The entire wear experiment lasted 170 days, with significant faults emerging in the support bearings in the later stages. Following data acquisition, preprocessing was performed, and the rotational error signals were discretized. Statistical analysis indicated that the spindle’s rotational error ranged from 5 μm to 14.5 μm. During discretization, error values were rounded to the nearest 0.5 μm, resulting in 20 error categories. The choice of 0.5 μm as the discretization step size is grounded in both engineering practice and established literature. From an engineering perspective, spindle rotational error directly determines machining accuracy, and a common strategy for precision enhancement is error compensation, where the measured error is used to adjust the tool path in real time. For this compensation to be effective, the error granularity must be comparable to or finer than the machining tolerance. The 0.5 μm step provides sufficient granularity to support micron-level error compensation—a typical requirement in high-precision machining operations. A finer step, such as 0.1 μm, would increase the number of categories to 95, transforming the classification task into a regression problem. This would not only increase model complexity but also introduce significant label noise, due to the measurement accuracy limitations of the sensors, making such fine-grained distinctions unreliable. Conversely, a coarser step, such as 1.0 μm, would result in only 10 categories, potentially losing discriminative information necessary for precise compensation. Furthermore, the 0.5 μm discretization setting follows the same configuration adopted in a recent study on spindle rotation error prediction [26]. Therefore, 0.5 μm was selected as the optimal step size that balances classification granularity, measurement precision, and practical engineering requirements.

In the experiment, two subsets were selected from each category, yielding a total of 40 subsets for subsequent analysis. Each subset contained 200,000 × 3 raw data points, corresponding to three vibration sensors. This study utilized the vibration signals from these three sensors along the *Z*-axis as the experimental data. To reflect real-world industrial application scenarios, the dataset was divided chronologically: the first 70% of data points from each signal segment formed the training set, and the remaining 30% constituted the test set. During training, a data augmentation method employing overlapping sampling was used to expand the sample size. Each sample had a length of 1024 data points with a sliding step size of 320. Consequently, the number of samples per error category increased to 870, resulting in a total training set size of 17,400 samples. No data augmentation was applied to the test set, which contained a total of 2320 test samples. Meanwhile, the min-max normalization method and STFT are employed for data processing. It is important to note that the chronological division (70% training/30% test) was performed prior to the overlapping sampling operation; thus, no data from the test set was used in training, and there is no risk of data leakage.

### 4.2. Hyperparameters and Comparative Methods

To comprehensively evaluate the performance of the proposed MFAFENet, this study selected a variety of representative deep learning models for comparative analysis. All models were trained and tested using the same dataset and preprocessing pipeline. The core hyperparameters involved in the experiment and the structures of the comparative methods are uniformly described in Table 2 and Table 3 to ensure the fairness and reproducibility of the comparative study.

#### 4.2.1. Hyperparameter Settings

The training of all models adopted the unified hyperparameter configuration shown in Table 2. This configuration draws on a benchmark study [34] in the field to maintain consistency.

#### 4.2.2. Comparative Models

The eight selected comparative models cover classic baselines, temporal models, multi-scale models, and mainstream attention mechanism models. Their core architectural characteristics are summarized in Table 3.

Among them, CNNs serve as basic controls to evaluate the feature information extraction capability of CNNs without residual connections and attention mechanisms. CBiLSTM, as an excellent temporal prediction model, is used to investigate whether combining spatio-temporal feature information modeling is suitable for the spindle rotational error classification task. MSCNN represents a main improvement direction for CNNs, employing a feature information fusion method that directly concatenates the outputs of parallel multi-branch convolutions (e.g., with 3 × 3, 5 × 5, 7 × 7 kernels). The comparison with it aims to highlight the advancement of the proposed MFAFConv layer, which can dynamically adjust the weights of scale branches. ResNet is the backbone network of the proposed method, serving as the baseline model to isolate and prove the independent contributions of MFAFConv and the ECA module. AWResNet and CResNet are two widely used attention mechanism variants currently, which recalibrate feature information through SENet (channel attention) and CBAM (channel and spatial hybrid attention), respectively. Using them for comparison aims to verify the superiority of the MFAFConv + ECA combination structure designed in this paper for the spindle rotational error classification task. It should be noted that the architecture and parameters of ResNet are consistent with Table 1, with the difference being that ResNet employs depthwise separable convolution (the number of groups equals the number of input channels), whereas MFAFENet utilizes the MFAFConv and ECA modules. LiConvFormer is a recent deep learning architecture that combines a separable multiscale convolution block for local feature extraction and a broadcast self-attention block for global fine-grained feature capture, representing the emerging trend of hybrid CNN-Transformer models for vibration-based condition monitoring. MobileNetV2 is a lightweight network built on inverted residuals with linear bottlenecks, widely used in resource-constrained scenarios due to its efficient architecture.

In addition, The performance of all models is uniformly evaluated using classification accuracy as the metric, calculated by the formula:

(11) $$Accuracy=\frac{N_{correct}}{N_{total}}\times 100\%$$

where $N_{correct}$ is the number of test samples correctly classified by the model, and $N_{total}$ is the total number of test samples.

#### 4.2.3. Comparative Experimental Results Analysis

Five independent trials were conducted for each model. Table 4 presents the average test accuracy and standard deviation across different methods. The proposed MFAFENet model achieves an average test accuracy of 93.37% (standard deviation ±0.29) across five independent trials, outperforming all comparative models. Specifically, MFAFENet shows an improvement of 5.14% over the baseline CNN model (88.23%) and 3.60% over the temporal modeling-capable CBiLSTM model (89.77%). In comparisons with multi-scale architectures, MFAFENet surpasses the traditional branch fixed-weight MSCNN model (90.65%) by 2.72%, demonstrating the effectiveness of its adaptive multi-scale fusion mechanism. Furthermore, when compared to enhanced residual networks integrated with attention mechanisms, MFAFENet achieves average improvements of 0.65% over AWResNet (92.72%) and 2.11% over CResNet (91.26%). Notably, the standard deviations of both MFAFENet (±0.29) and AWResNet (±0.28) are very small, indicating that the performance of each model is stable and the observed 0.65% improvement, while modest, is consistent and reproducible across five independent trials. Compared with lightweight models, MFAFENet outperforms LiConvFormer (91.88%) by 1.49% and MobileNetV2 (92.13%) by 1.24%. These quantitative results fully validate that the combined design of the MFAFConv and the ECA module in MFAFENet can more effectively extract and enhance discriminative feature information from vibration signals, thereby achieving superior predictive performance in the spindle rotational error classification task. Integration with CNC control systems can be achieved by connecting vibration sensors to a data acquisition module, transmitting signals to an edge device running MFAFENet, and outputting predicted rotational errors to the CNC controller for real-time error compensation.

In addition to accuracy, we also evaluate model complexity in terms of parameter count, computational complexity, and inference time, as shown in Table 4. The inference time reported for each model is the total time required to process all 2320 test samples on an RTX 2060 SUPER GPU. MFAFENet achieves the highest accuracy (93.37%) while using only 156.14 k parameters, which is far fewer than ResNet (11.19 M) and AWResNet (11.80 M). Compared to lightweight models, MFAFENet also uses significantly fewer parameters than MobileNetV2 (2.25 M) and LiConvFormer (744.72 k). This significant reduction is mainly attributed to the use of depthwise separable convolution in each branch of the MFAFConv module. In terms of computational complexity, MFAFENet requires only 30.01 M per forward pass, which is much lower than ResNet (3.23 G) and AWResNet (3.23 G), as well as lower than LiConvFormer (196.43 M) and MobileNetV2 (66.82 M). Regarding inference time, MFAFENet completes inference on all 2320 test samples in 0.57 s, which is faster than ResNet (1.39 s) and AWResNet (1.46 s), while MobileNetV2 achieves 0.34 s and LiConvFormer achieves 0.38 s, both faster than MFAFENet due to their lightweight design. For real-time monitoring, the inference time of 0.57 s for 2320 samples translates to approximately 0.25 ms per sample, which is well below the typical sampling interval of vibration signals (0.05–0.1 ms at 10–20 kHz). This indicates that MFAFENet can process incoming vibration data in real time without causing latency bottlenecks. Furthermore, with only 156.14 k parameters, the model can be easily deployed on resource-constrained edge computing devices commonly used in factory environments. These results demonstrate that the proposed MFAFENet strikes an excellent balance between predictive accuracy and computational efficiency, making it suitable for real-time spindle rotational error monitoring in practical industrial applications.

**Figure 7:**
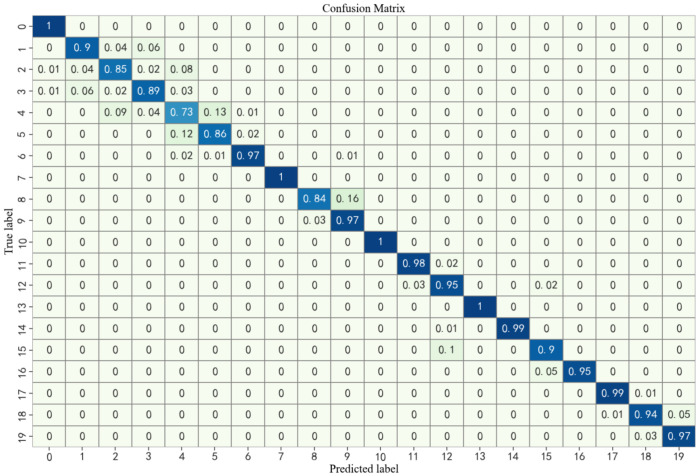
Confusion matrix of MFAFENet on the spindle rotational error classification task. Rows represent predicted labels and columns represent true labels. Diagonal values indicate correct classification rates, showing that most misclassifications occur between adjacent error categories.

Furthermore, the confusion matrix was applied to evaluate the classification performance of the model across individual classes. Figure 7 presents the confusion matrix obtained from the MFAFENet models in the task of spindle rotational error classification. In the matrix, rows correspond to the predicted class labels, while columns denote the true class labels. The value located at the intersection of row i and column j indicates the fraction of instances actually belonging to class j that were predicted as class i. Analysis of the confusion matrix reveals that MFAFENet achieves prediction accuracies exceeding 90% across 15 categories. The majority of categories with a prediction accuracy below 90% have a close rotational error. For example, for categories with a true label of 8 (9 μm), 16% predicted 9 (9.5 μm), which is relatively close to the true value. This occurs because the rotational errors between such adjacent categories are very small, resulting in highly similar vibration signal characteristics and small inter-class differences. Such prediction outcomes are generally acceptable in practical applications.

#### 4.2.4. Ablation Study

The primary innovation of MFAFENet lies in developing the MFAFConv module to replace standard depthwise separable convolution and incorporating the ECA module. To validate the effectiveness of the MFAFConv and ECA modules, an ablation study was conducted. The experimental results are presented in Table 5. The results indicate that both proposed core modules—MFAFConv and ECA—contribute significantly to the model’s performance improvement. Specifically, based on the benchmark ResNet model (90.22%), replacing the standard depthwise separable convolution solely with the MFAFConv module elevates the average test accuracy to 92.58%, achieving an absolute gain of 2.36%. This validates that the proposed adaptive multi-scale feature information fusion mechanism can more effectively extract critical discriminative information from vibration signals. Meanwhile, the ResNet + ECA baseline achieves 91.52% accuracy, which is 1.3% higher than the original ResNet (90.22%), confirming that the ECA module alone also contributes to performance improvement by recalibrating channel-wise feature responses. Further, incorporating the ECA module into the MFAFConv-integrated model to construct the complete MFAFENet boosts the accuracy to 93.37%, yielding an additional performance improvement of 0.79% compared to the version with only MFAFConv. This outcome demonstrates that the ECA module, by modeling dependencies along the channel dimension and adaptively recalibrating feature channels, effectively enhances the model’s feature representation capability. Furthermore, MFAFENet achieves 93.37% accuracy, which is 1.85% higher than ResNet + ECA (91.52%), demonstrating that the adaptive branch weighting mechanism in MFAFConv is effective in further enhancing feature representation. Ultimately, MFAFENet achieves an overall performance gain of 3.15% relative to the original ResNet baseline, providing comprehensive and quantitative evidence for the efficacy and necessity of the MFAFConv and ECA module designs.

#### 4.2.5. Selection of Multi-Scale Branch Number M

**Figure 8:**
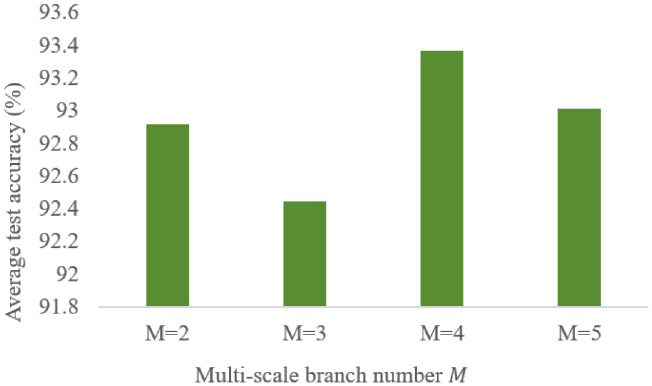
Comparison of prediction results with different branch numbers.

The number of multi-scale branches M is a critical hyperparameter in the MFAFENet model. A small number of branches may be insufficient to extract adequate multi-scale feature information, while an excessively large M can significantly increase model parameters and introduce redundant feature information. In this study, M is set to 2, 3, 4, and 5. Under the same network architecture and parameter settings, comparative experiments were conducted to evaluate the impact of different branch numbers. The experimental results are shown in Figure 8. The experimental results indicate that model performance varies with different M values. When M=4, MFAFENet achieves the highest average test accuracy of 93.37%, outperforming M=2 (92.92%) by 0.45% and M=3 (92.45%) by 0.92%. Notably, the model with M=3 exhibits slightly lower accuracy than that with M=2, suggesting that simply increasing the number of branches does not guarantee performance improvement; inappropriate branch configuration may introduce redundant or interfering information. When M = 5 (with kernel sizes 3, 5, 7, 9, 11), the accuracy drops to 93.01% and the parameter count increases to 226.54 k. Compared to M = 4 (156.14 k parameters, 93.37% accuracy), M = 5 introduces 70.4 k additional parameters but results in a 0.36% accuracy decrease, indicating that adding larger kernels not only increases model complexity but also introduces interfering information that degrades performance. When M=4, the model achieves optimal performance, indicating that four parallel branches with kernel sizes of 3×3, 5×5, 7×7, and 9×9 can effectively capture multi-scale time-frequency feature information from vibration signals while maintaining a reasonable balance between feature information diversity and model complexity. Therefore, M=4 is selected as the optimal hyperparameter configuration for MFAFENet.

#### 4.2.6. Comparison with Common Multi-Scale Fusion Methods

As discussed earlier, existing multi-scale convolutional structures employ multiple branches, each using convolutional kernels of different sizes to extract feature information, and finally fuse the feature information from these branches through concatenation or summation. To validate the superiority of the proposed adaptive feature information fusion approach, comparative experiments were conducted against three commonly used multi-scale feature information extraction methods. Model I divides the convolution in ResNet into M groups (M = 4), with m∈[1,M], where the kernel size of m-th group is (2m+1). The feature information from the M groups are concatenated, and an ECA module is added after the convolution. Model II removes the branch weighting mechanism from MFAFENet, directly using the element-wise summed feature $X_{fused}$ as the output of the convolutional layer, followed by an ECA module. Inception-based baseline replaces the MFAFConv module with the standard Inception block, using parallel convolutions with kernel sizes 1 × 1, 3 × 3, 5 × 5, and max pooling while retaining the ResNet backbone and ECA module. The experimental results are presented in Table 6.

The experimental results show that multi-scale feature information fusion model I achieves an average test accuracy of 92.20%, model II achieves 91.90%, and the Inception-based baseline achieves an average test accuracy of 91.51%, while the proposed MFAFENet attains an accuracy of 93.37%. Compared to model I and model II, MFAFENet achieves significant performance improvements of 1.17% and 1.47%, respectively. Compared to the Inception-based baseline, MFAFENet achieves a performance improvement of 1.86%. This quantitative comparison fully demonstrates that conventional multi-scale fusion methods, which rely on fixed concatenation or summation strategies, cannot dynamically adjust the importance of different scale branches based on the input signal content, thereby limiting their feature representation capability. In contrast, the proposed MFAFConv module adaptively assigns weights to different scale branches through a branch attention mechanism, dynamically aggregating feature information from various receptive fields, and thus captures key discriminative feature information related to spindle rotational error more precisely. In summary, the adaptive feature information fusion mechanism exhibits clear performance advantages over conventional multi-scale fusion methods, validating the effectiveness and superiority of the proposed design.

#### 4.2.7. Multi-Sensor Effectiveness Validation Experiment

**Figure 9:**
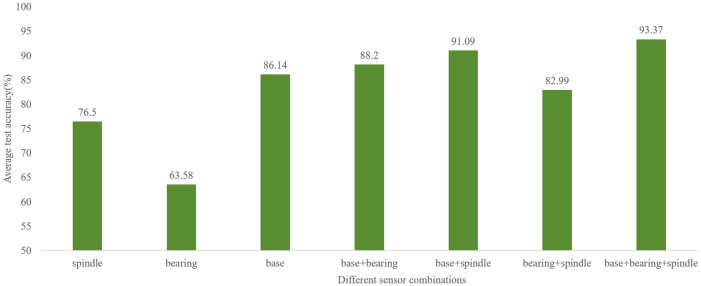
Comparison of prediction results with different data sources.

To validate the effectiveness of multi-sensor data fusion, comparative experiments were conducted. Single-sensor data from the spindle end, bearing end, and base end, as well as two-sensor combinations (base + bearing, base + spindle, bearing + spindle) and the three-sensor fusion, were used as inputs. The experimental results are shown in Figure 9. The experimental results demonstrate that the multi-sensor data fusion strategy significantly outperforms all single-sensor and two-sensor configurations. Among the single-sensor inputs, the base end sensor achieves the highest accuracy of 86.14%, followed by the spindle end sensor at 76.50%, while the bearing end sensor yields the lowest accuracy of 63.58%. This indicates that vibration signals collected from different mounting positions exhibit varying sensitivities to spindle rotational errors, with the base end providing the most informative single-source measurements. Among the two-sensor combinations, base + spindle achieves the highest accuracy of 91.09%, followed by base + bearing (88.20%) and bearing + spindle (82.99%). The base + spindle combination performs notably better than base + bearing, suggesting that the spindle end sensor provides complementary information to the base sensor that is more relevant to rotational error classification. All two-sensor combinations outperform their constituent single sensors, demonstrating the benefit of multi-sensor collaboration even with only two sensors.

Notably, the proposed three-sensor fusion approach, integrating signals from all three positions, achieves an average test accuracy of 93.37%—substantially higher than any individual sensor or two-sensor configuration. Specifically, compared to the best-performing single sensor (base end, 86.14%), multi-sensor fusion yields an absolute improvement of 7.23%. Compared to the best-performing two-sensor combination (base + spindle, 91.09%), the improvement is 2.28%. These quantitative results provide strong evidence for the effectiveness of multi-sensor collaboration. The complementary information captured by sensors at different mounting positions enables a more comprehensive characterization of spindle operational states, effectively mitigating the limitations of single-source measurements that are susceptible to transmission path effects, localized interference, or insufficient sensitivity. The substantial performance gains validate that the multi-sensor input strategy employed in MFAFENet is essential for achieving high-precision spindle rotational error classification. For practical industrial deployment, we recommend installing sensors at all three positions (base end, spindle end, and bearing end) to achieve the highest prediction accuracy. If cost or installation space is limited, the base end and spindle end combination provides a good trade-off between accuracy and hardware cost.

#### 4.2.8. Sensitivity Analysis of Reduction Factor β

The reduction factor β in the MFAFConv module controls the compression ratio in the branch attention mechanism. The first convolutional layer compresses the channel descriptor from C to C/β channels. This compression serves two purposes: it reduces the number of parameters in the attention module, improving computational efficiency; it forces the network to learn a compact representation of inter-branch relationships, which helps avoid overfitting. To evaluate the impact of β on model performance and to justify our choice, we conducted a sensitivity analysis with β values of 2, 4, and 8. All other hyperparameters were kept identical to those in Table 2. The experimental results are presented in Table 7. The model achieves the best performance (93.37%) when β = 4. When β = 2, the compression ratio is smaller (C → C/2), leading to a larger number of parameters (210.66 k) and slightly lower accuracy (92.82%). When β = 8, the compression is more aggressive (C → C/8), resulting in fewer parameters (128.88 k) but a noticeable accuracy drop to 92.59%. This indicates that excessive compression may cause information loss in the branch attention mechanism, degrading classification performance. Therefore, β = 4 is selected as the optimal value for all experiments in this study, as it achieves the best trade-off between accuracy and model efficiency.

## 5. Conclusions

This paper proposes a novel deep learning model, MFAFENet, for CNC machine tool spindle rotational error classification based on multi-sensor collaboration and multi-scale feature information adaptive fusion. The model first employs STFT to transform multi-sensor vibration signals into time-frequency representations, then utilizes the proposed MFAFConv module with branch attention mechanism to adaptively fuse multi-scale feature information from different receptive fields, and finally incorporates the ECA module to recalibrate channel-wise feature responses. Extensive experiments conducted on a spindle reliability test bench demonstrate that: (1) MFAFENet achieves an average test accuracy of 93.37%, significantly outperforming CNN, CBiLSTM, MSCNN, ResNet, AWResNet, CResNet, LiConvFormer, and MobileNetV2 with improvements ranging from 0.65% to 5.14%; (2) Ablation studies validate that both MFAFConv and ECA modules contribute substantially to performance gains, yielding absolute improvements of 1.85% and 0.79%, respectively, and the full MFAFENet achieves a 3.15% overall gain over the ResNet baseline; (3) Comparative experiments confirm that the proposed adaptive multi-scale fusion mechanism surpasses conventional fixed-weight multi-scale fusion methods by 1.17–1.86%; (4) Multi-sensor effectiveness validation shows that fusing signals from three mounting positions achieves 7.23% higher accuracy than the best single-sensor configuration and 2.28% higher than the best two-sensor combination, demonstrating the necessity of multi-sensor collaboration; (5) Hyperparameter analysis determines M=4 as the optimal number of multi-scale branches and β=4 as the optimal reduction factor, providing the best balance between feature information diversity and model complexity. In summary, MFAFENet effectively addresses the limitations of existing methods in modeling branch interrelationships and channel-wise dependencies, establishing an end-to-end mapping between vibration signals and rotational errors with superior discriminative capability for this challenging non-fault classification task characterized by small inter-class distances.

For future work, we plan to explore the application of large language model techniques for spindle rotational error prediction, leveraging their powerful semantic understanding and cross-modal representation capabilities to further enhance prediction performance and model interpretability. In addition, all experiments in this study were conducted on a single internal dataset, as there is currently no publicly available benchmark dataset specifically designed for spindle rotational error classification. To address this limitation, we explicitly plan to construct and release a public benchmark dataset for this task in our future work, which will facilitate fair comparisons and validation of different methods in this emerging field.

## Figures and Tables

**Table 1:** Hyperparameters of MFAFENet.

Layer	Kernel Size	Input Dimension	Output Dimension
Conv + BN + ReLU	3	3 × 33 × 33	16 × 17 × 17
Improved residual block1 × 2	3, 5, 7, 9	16 × 17 × 17	16 × 17 × 17
Improved residual block2 × 2	3, 5, 7, 9	16 × 17 × 17	32 × 17 × 17
Improved residual block3 × 2	3, 5, 7, 9	32 × 17 × 17	64 × 17 × 17
Improved residual block4 × 2	3, 5, 7, 9	64 × 17 × 17	128 × 17 × 17
GAP	-	128 × 17 × 17	128 × 1 × 1
Fully connected	-	128 × 1 × 1	20

**Table 2:** General hyperparameter settings for model training.

Hyperparameter	Set Value	Description
Batch size	64	Balances training stability, memory usage, and computational efficiency.
Optimizer	Adam	Integrates adaptive learning rate adjustment, $\beta_{1}$ = 0.9.
Initial learning rate	0.001	The starting learning rate for training all models.
Learning rate decay	0.1	The learning rate decays by 0.1 at epochs 30 and 60.
Weight decay	1 × 10^−5^	Prevents model overfitting.
Training epochs	100	The total number of training iterations.

**Table 3:** Comparative models and their core characteristics.

Model	Core Characteristics and Comparison Purpose
CNN	Enhanced CNN baseline.
CBiLSTM [34]	Temporal feature model. Tests the effectiveness of explicitly modeling temporal dependencies.
MSCNN [26]	Fixed multi-scale model. Compare with traditional multi-scale structure.
ResNet [24]	Benchmark residual model. Serves as the backbone network for MFAFENet, used to evaluate the contribution of the proposed modules. The number of output channels for the four residual blocks is 16, 32, 64, and 128, respectively.
AWResNet [5]	Channel attention model. Integrates the Squeeze-and-Excitation module, used to compare the performance of the ECA module.
CResNet [35]	Hybrid attention model. Integrates the Convolutional Block Attention Module, used for comparative verification of the effectiveness of the proposed overall architecture.
LiConvFormer [36]	A lightweight framework that uses a separable multiscale convolution block to extract multi-local features and a broadcast self-attention block to capture global fine-grained features.
MobileNetV2 [37]	A lightweight network built on inverted residuals with linear bottlenecks.

**Table 4:** Comparative experimental results.

Model	Complexity	Inference Time	Parameters	Average Test Accuracy
CNN	16.95 M	0.12 s	2.33 M	88.23 ± 0.98
CBiLSTM	6.4 M	0.14 s	977.24 k	89.77 ± 0.28
MSCNN	851.44 M	0.44 s	16.98 M	90.65 ± 0.26
ResNet	3.23 G	1.39 s	11.19 M	90.22 ± 0.38
AWResNet	3.23 G	1.46 s	11.80 M	92.72 ± 0.28
CResNet	3.24 G	1.75 s	11.27 M	91.26 ± 0.69
LiConvFormer	196.43 M	0.38 s	744.72 k	91.88 ± 0.33
MobileNetV2	66.82 M	0.34 s	2.25 M	92.13 ± 0.53
MFAFENet	30.01 M	0.57 s	156.14 k	93.37 ± 0.29

**Table 5:** Ablation study results.

Model	Average Test Accuracy
ResNet	90.22 ± 0.38
ResNet + MFAFConv	92.58 ± 0.59
ResNet + ECA	91.52 ± 0.35
ResNet + MFAFConv + ECA (MFAFENet)	93.37 ± 0.29

**Table 6:** Experimental results of comparison with common multi-scale fusion methods.

Model	Average Test Accuracy
Multi-scale feature information fusion model I	92.20 ± 0.43
Multi-scale feature information fusion model II	91.90 ± 0.57
Inception-based baseline	91.51 ± 0.53
ResNet + MFAFConv + ECA (MFAFENet)	93.37 ± 0.29

**Table 7:** Experimental results of reduction factor β sensitivity analysis.

*β* Value	Parameters	Average Test Accuracy
*β* = 2	210.66 k	92.82 ± 0.20
*β* = 4	156.14 k	93.37 ± 0.29
*β* = 8	128.88 k	92.59 ± 0.33

## Data Availability

The data presented in this study are available on request from the corresponding author.
